# Transhepatic arterial infusion chemotherapy using a combination of miriplatin and CDDP powder versus miriplatin alone in the treatment of hepatocellular carcinoma: a randomized controlled trial

**DOI:** 10.1186/s12885-017-3320-7

**Published:** 2017-05-10

**Authors:** Kenya Kamimura, Takeshi Suda, Takeshi Yokoo, Hiroteru Kamimura, Tsutomu Kanefuji, Atsunori Tsuchiya, Masaaki Takamura, Hirokazu Kawai, Nobuo Waguri, Satoshi Yamagiwa, Shuji Terai

**Affiliations:** 1 Division of Gastroenterology and Hepatology, Graduate School of Medical and Dental Sciences, Niigata University, Niigata, 951-8510 Niigata Japan; 2 Department of Gastroenterology and Hepatology, Uonuma Institute of Community Medicine, Niigata Medical and Dental Hospital, 4132 Urasa, Minami-Uonuma, 949-7302 Niigata Japan; 3 Department of Gastroenterology and Hepatology, Niigata City General Hospital, Niigata, 950−1197 Niigata Japan

**Keywords:** Hepatocellular carcinoma, Interventional radiology, Miriplatin, Cisplatin powder, Phase II clinical trial

## Abstract

**Background:**

Based on promising results from a Phase I study of hepatic arterial infusion chemotherapy using a combination of miriplatin and cisplatin powder (DDP-H) for unresectable hepatocellular carcinoma (UMIN-CTR000003541), a multicenter, open-label, randomized phase II study was conducted to evaluate the efficacy and safety of the combination therapy versus miriplatin monotherapy.

**Methods:**

Nineteen patients, five and fourteen Barcelona-Clinic Liver Cancer staging classification A and B cases, respectively, were randomly assigned to receive either miriplatin monotherapy (*n* = 9) or miriplatin/DDP-H combination therapy (*n* = 10). DDP-H and/or miriplatin were administered through the hepatic arteries supplying the lobes of the liver containing tumors, and progression free survival was analyzed as a primary end point in addition to other secondary endpoints. The corresponding therapy was repeated unless disease progression or severe adverse events were recorded.

**Results:**

The monotherapy or combination therapy was performed for 15 or 36 sessions in total, respectively. Although there were no significant differences between the two groups for treatment intervals (*p* = 0.96) or the dose of miriplatin used in each session (*p* = 0.99), the progression free survival and overall disease control rate were significantly better in the combination therapy group (91 vs 423 days, *p* = 0.025; 40.0 vs 77.8%, *p* = 0.0025, respectively). Consistent with these observations, a trend of a significantly slower increase in des-γ-carboxyprothrombin was observed, and the number of treatment sessions was nearly significantly larger in the combination therapy group (*p* < 0.0001, *p* = 0.057, respectively). Conversely, the median survival time did not show a significant difference (706 days, monotherapy vs 733 days, combination therapy; *p* = 0.40). A significant decrease in cholinesterase was observed during the course of treatment only in patients receiving combination therapy (*r* = −0.86, *p* < 0.0001). A few cases in both arms showed hematological and/or non-hematological toxicities that were categorized as grade 1 (NCI-CTCAE).

**Conclusions:**

The higher disease control effects with the combination of miriplatin and DDP-H indicate that it is a promising alternative treatment for cases with multiple HCCs, especially for those that can tolerate the treatment without experiencing a reduction in hepatic reserve.

**Trial registration:**

This study was registered on 1 January 2012 with the University Hospital Medical Information Network Clinical Trials Registry (http://www.umin.ac.jp/ctr/index.htm, UMIN000004691).

## Background

Hepatocellular carcinoma (HCC) is the third most common cause of cancer-related death and the number of HCC cases is increasing globally [1, 2]. Various therapeutic options have been developed that focus on tumor stage and hepatic functional reserve [3, 4], including transarterial treatments such as transarterial chemoembolization (TACE), transarterial oily chemoembolization (TOCE), and hepatic arterial infusion chemotherapy (HAIC). These treatments take advantage of the fact that HCCs are fed to a greater extent by arterial blood than the surrounding liver parenchyma. Based on the fact that TOCE showed anti-tumor effects similar to TACE, especially in patients with multiple HCCs and a lower hepatic reserve [5], miriplatin, a third-generation platinum derivative with a lipophilic moiety that forms a suspension with lipiodol, was developed and approved for clinical use in Japan as a novel chemotherapeutic agent for use in TOCE [6, 7]. On the other hand, HAIC can cover a larger area of the liver without reducing hepatic arterial flow, the combination of HAIC with TOCE [5, 8, 9] may improve treatment efficacy but retain an unfavorable toxicity profile. In terms of the chemotherapeutic agent used in HAIC, promising results from a multicenter phase II study in patients with unresectable HCC using cisplatin (CDDP), a first-generation platinum agent, have been reported [10]. Because the chemotherapeutic action of platinum agents depends on their concentration, HAIC has been widely applied in Japan by utilizing a CDDP powder (DDP-H, IA-call®; Nippon Kayaku Co., Ltd), as it has been reported to provide the highest concentration of CDDP [8, 10, 11]. Given these advances in the development of new chemotherapeutics, we conducted a phase I study on the combination therapy of miriplatin-TOCE and DDP-H-HAIC and demonstrated that miriplatin and DDP-H can be administered in combination up to the maximum tolerated dose of each drug without additional adverse events. Here, we report the safety and efficacy profiles of the combination therapy based on the results of the subsequent phase II clinical trial.

## Methods

### Study design

This multicenter, open-label, randomized phase II trial was designed to compare the efficacy of miriplatin-TOCE monotherapy and miriplatin-TOCE/DDP-H-HAIC combination therapy and was conducted across 3 Japanese institutions. This study was approved by each Institutional Review Board and was registered on the University Hospital Medical Information Network Clinical Trials Registry (http://www.umin.ac.jp/ctr/index.htm, UMIN000004691). Written informed consent for publication of their clinical details and/or clinical images was obtained from the patient. A copy of the consent form is available for review by the Editor of this journal. The study protocol conformed to the ethical guidance of the 1975 Declaration of Helsinki.

### Patient selection

Patients with HCC were considered eligible for the study if they fulfilled the following criteria: histologically and/or clinically diagnosed HCC: no indication or consent for surgical resection, radiofrequency ablation, radiation therapies, or liver transplantation according to the Japanese guidelines [12]; 20–80 years of age; at least one tumor larger than 10 mm that could be measured by CT and/or MRI as an enhanced nodule; an Eastern Cooperative Oncology performance status of 0–2; preserved organ function using indicators within the liver (Child–Pugh, score ≤ 8; total bilirubin, ≤3.0 mg/dl; albumin, ≥3.0 g/dl), blood (neutrophils, ≥1500/mm^3^; platelets, ≥50,000/mm^3^; hemoglobin, ≥8.0 g/dl), and kidneys (creatinine clearance, ≥50 ml/min adjusted for 1.73 m^2^ of body surface area by measuring creatinine concentrations in the blood and urine collected for a day); serum amylase, ≤324 IU/dl; and an interval of 4 weeks or longer after a previous treatment for HCC. Patients with the following features were considered ineligible: active infectious disease; massive pleural effusion and/or ascites refractory to treatments; active bleeding from the gastrointestinal tract; active concomitant cancer with invasive characteristics; severe mental disorder or hepatic encephalopathy; history of allergic reaction to iodine phase contrast and/or platinum agents; pregnant or lactating female; ongoing interferon therapy; difficulty with oral food intake; and other serious conditions judged to be inadequate by responsible physicians.

### Method of administration

Under a local anesthesia, a 3 to 5-Fr hook or cobra-shaped catheter (GADELIUS Medical K.K., Japan; Terumo Corporation, Japan; Hanaco Medical Co., Ltd., Japan,) was introduced from the right femoral artery depending on arterial curvatures as anticipated by CT images and was anchored at the celiac trunk or common hepatic artery for the further insertion of 1.8/2.4-Fr (Carnelian Marvel, Tokai Medical Products, Japan) or 2.2/2.9-Fr (Carnelian ER, Tokai Medical Products, Japan) micro catheter, which was coaxially aligned with a 0.016-in. Radifocus™ wire (Terumo Corp., Tokyo, Japan). Patients were randomly assigned to receive either miriplatin monotherapy (TOCE) or miriplatin/DDP-H combination therapy (TOCE + HAIC). In a case of the TOCE group, a tip of the micro catheter was advanced as near as possible to the tumors, and 20 mg/ml of miriplatin dissolved in lipiodol was administered up to 120 mg/body, which was divided if the tumors were supplied via multiple vessels. For the TOCE + HAIC group, DDP-H was dissolved in saline at a concentration of 1.43 mg/ml and administered as HAIC at a rate of 3 mg/min through the hepatic arteries supplying the lobes of the liver containing tumors. If the tumors were located in the posterior, anterior, or both segments, a tip of the micro catheter was placed at the right hepatic artery, while the tip was placed at the left hepatic artery if the tumors were localized in the lateral, medial, or both segments. If the tumors were spread in both lobes, the tip of the catheter was positioned at the proper hepatic artery. After the administration of DDP-H, a successive TOCE was performed using miriplatin as described above. The dose of DDP-H was determined based on renal function. When creatinine clearance was 100 ml/min or less, DDP-H was infused up to the value of creatinine clearance [13]. For example, when creatinine clearance was 70 ml/min, 70 mg of DDP-H was infused in total. The highest dose of DDP-H was 100 mg/body. The corresponding treatment of each enrolled arm was repeated when the following CT/MRI study showed a stable disease/partial response or when hypervascular nodules appeared again in a case achieving complete response.

### Efficacy and safety assessment

The anti-tumor response was evaluated by comparing CT/MRI images obtained before treatment and 3 months after treatment. The evaluation was performed in accordance with the modified Response Evaluation Criteria in Solid Tumors guideline [14]. The disease control rate (DCR) was defined as the percentage of treatment sessions in which anti-tumor effects were judged to be either a complete response, partial response or stable disease. The National Cancer Institute Common Terminology Criteria for Adverse Events (NCI-CTCAE) version 4.0 was used to assess toxicity. The corresponding treatment of the enrolled arm was repeated until disease progression and/or adverse events of grade ≥ 3 non-hematological or grade ≥ 4 hematological toxicities were observed. To evaluate the impact of each treatment on functional hepatic reserve, serum biomarkers were analyzed prior to each treatment session and 3 months after the final treatment. The values were analyzed after conversion to a percentage of the value prior to the initial treatment. We assessed three tumor markers, α-fetoprotein (AFP), des-γ-carboxy prothrombin (DCP), and the percentage of fucosylated AFP versus total AFP (L3%). AFP and DCP were converted first to a log scale value and then to a percentage value against the value prior to the initial treatment, while L3% was analyzed without any conversion. The primary endpoint was progression free survival (PFS), and DCR, overall survival (OS), the number of repetitive treatment sessions, the interval between the sessions, adverse events, and the dose of administered miriplatin were calculated at the time of the primary endpoint.

### Statistical analysis

PFS and OS along with the hazard ratio and 95% confidence interval (CI) for the median time to the event were assessed using the Kaplan–Meier method and log-rank test. Numerical or categorical parameters were analyzed using the Mann–Whitney–Wilcoxon or Fisher’s exact test, respectively. Spearman correlation coefficients were calculated to clarify a specific alteration in a biomarker or other numerical properties during the course of treatment. All statistical analyses were performed using GraphPad Prism 6 (San Diego, CA, USA), and a 2-sided *p* value of ≤0.05 was considered to be statistically significant.

## Results

### Patient characteristics

**Fig. 1:**
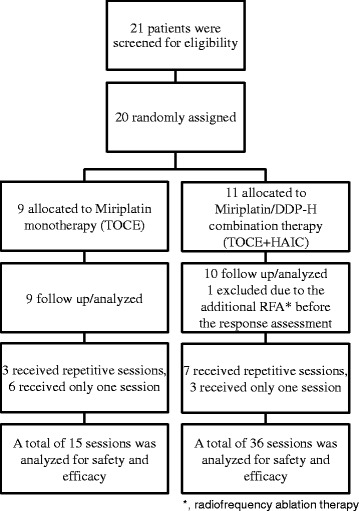
A consort diagram of the study

From 2010 to 2013, a total of 21 patients were screened for eligibility (Fig. 1). Based on a pretreatment evaluation (see Methods), 20 eligible patients were enrolled in this study and randomly assigned to one of the 2 study arms, either the TOCE monotherapy or TOCE + HAIC combination therapy. Among the 11 patients allocated to the TOCE + HAIC, one case was excluded from further follow-up because radio frequency ablation therapy was performed on the target before the first efficacy evaluation. The baseline characteristics of the patients showed no statistically significant differences between the 2 arms in terms of age, gender, performance status, etiology of background diseases, presence or absence of liver cirrhosis, Child-Pugh grade, tumor size, tumor number, TNM stage, stage of Barcelona-Clinic Liver Cancer staging classification (BCLC), or treatment history using DDP-H or TACE (Table 1).

**Table 1:** Baseline characteristics

Items		TOCE	TOCE + HAIC	Provability
		*n* = 9	*n* = 10	
Age (years)	Median	81	72	0.22
	Range	55–85	36–84	
Gender	Female	2	5	0.09
	Male	7	5	
Performance status	0/1	8/1	10/0	0.47
Etiology	HBV infection	1	1	0.32
	HCV infection	7	5	
	Alcohol	0	1	
	NASH	0	1	
	Autoimmune hepatitis	0	1	
	Primary Biliary Cirrhosis	0	1	
	Unknown Etiology	1	0	
Cirrhosis (Yes/No)		9/0	10/0	1.00
Child-Pugh Grade	A/B/C	8/1/0	7/3/0	0.36
Size (mm)	Median	22	21.5	0.87
	Range	10–32	10–85	
Number	1	0	1	0.41
	≤3	4	1	
	>3	5	8	
TNM	I/II/III	0/3/6	0/5/5	0.55
BCLC	0/A/B/C/D	0/3/6/0/0	0/2/8/0/0	0.65
Previous Treatment	DDP-H	8	7	
	TACE	9	6	0.51

TOCE, transarterial oily chemoembolization; TACE, transarterial chemoembolization; HAIC, hepatic arterial infusion chemotherapy; NASH, non-alcoholic steatohepatitis; BCLC, Barcelona-Clinic Liver Cancer staging classification

### Treatment

The details of the treatments are summarized in Fig. 1 and Table 2. Three out of nine patients on TOCE (33%) and seven out of ten patients on TOCE + HAIC (70%) who received corresponding TOCE or TOCE + HAIC treatments repeatedly for fifteen or thirty-six sessions, respectively, approached a significant difference (*p* = 0.057). The intervals between sessions, 94.5 and 90.5 days in TOCE and TOCE + HAIC, respectively, showed no significant difference (*p* = 0.96). The dosage of miriplatin used in each session was not significantly different between the 2 arms (*p* = 0.99).

**Table 2:** Summary of treatments

Items		TOCE	TOCE + HAIC	Provability 95%CI
		*n* = 9	*n* = 10	
Number of Treatment Session	(1/2/3/4/5/6/7/8)	6/1/1/1/0/0/0/0	3/1/1/1/2/1/0/1	0.057
	Total	15	36	
	Median	1	3.5	
	Range	1–4	1–8	
Interval between Sessions (day)	Median	94.5	90.5	0.96
	Range	56–259	48–518	
DDP-H (mg/body)	Median	N/A	80	N/A
	Range	N/A	40–100	
Miriplatin (mg/body)	Median	62	62	0.99
	Range	40–120	16–120	
anti-tumor Effect^a^	CR	0 (0)	6 (3)	0.0025
	PR	0 (0)	7 (2)	
	SD	6 (3)	15 (5)	
	PD	9 (9)	8 (8)	
	Not evaluable	0	0	
	DCR (%)	40.0	77.8	
Disease Progression (day)	Median	91	423	0.025
	Range	29–322	34–1243	1.17–10.86
Overall Survival (day)	Median	706	733	0.40
	Range	62–1176	384–1827	0.54–4.54

^a^ anti-tumor effect was evaluated based on the modified Response Evaluation Criteria In Solid Tumors guideline. *CR* complete response, *PR* partial response, *SD* stable disease, *PD* progressive disease, *DCR* disease control rate, *HR* hazard ratio, *TOCE* transarterial oily chemoembolization, *TACE* transarterial chemoembolization, *HAIC* hepatic arterial infusion chemotherapy

### Efficacy

**Fig. 2:**
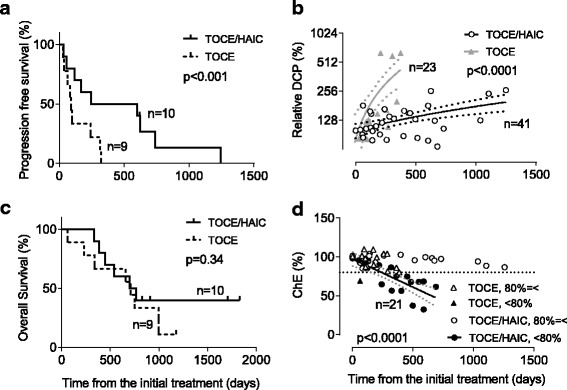
Efficacy and safety of the miriplatin-TOCE/DDP-H-HAIC combination therapy compared with the miriplatin-TOCE monotherapy. **a** Progression free survival time was significantly longer in the combination therapy group (*p* < 0.001; *continuous line*, combination therapy; *dotted line*, monotherapy). **b** Time dependent changes of des-γ-carboxyprothrombin (DCP) after each session. The relative DCP is a percentage of the log scale DCP value after treatments versus that obtained before the initial treatment (*open circles*, combination therapy (*p* = 0.034, *r* = 0.33); *closed triangles*, monotherapy (*p* < 0.0001, *r* = 0.80)). The continuous lines are the best hit lines accompanied with *dotted lines* showing 95% confidence intervals. The *black* and *grey* colors indicate the combination and monotherapies, respectively. **c** Kaplan-Meier estimates of overall survival were not significantly different between the two arms (*p* = 0.34, *continuous line*, combination therapy; *dotted line*, monotherapy). **d** Time-dependent changes in the serum concentrations of cholinesterase (ChE). Each value represents a percentage of the serum concentration of ChE versus the value obtained before the initial treatment. The *circles* and *triangles* represent the combination and monotherapies, respectively. The open mark shows the values from the cases, in which ChE never decreased to less than 80% of the values obtained before the initial treatments. In the closed marks, ChE did decrease to less than 80% of the initial values. The *dotted horizontal line* represents the 80% cutoff. The *continuous line* is the best hit line for the closed circular values with the *dotted diagonal line* that demonstrates 95% confidence intervals (*p* < 0.0001, *r* = −0.86). The number of cases in each arm was shown as “*n*=”

**Fig. 3:**
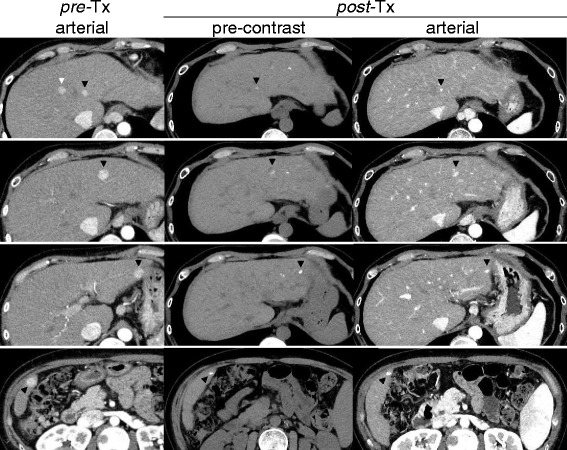
Representative computed tomography images from the study arm (case 4 in Table 4) treated with the miriplatin-TOCE/DDP-H-HAIC combination therapy. *Pre-*TX arterial: Multiple nodules were spread in both lobes and enhanced in the late arterial phase of a dynamic computed tomography study performed prior to the treatment (*black* and *white arrowheads*). *post*-Tx pre-contrast: These nodules were detected as high density spots of lipiodol without an injection of contrast medium three months after the eighth session of combination therapy (*black arrowheads*). *post*-Tx arterial: The high density spots were scarcely enhanced and markedly reduced in size (*black arrowheads*). One nodule, which is indicated by the *white arrowhead* in the *pre-*Tx arterial column, could not be detected at post-Tx either in the pre-contrast or in the arterial phase

The median periods of PFS were 91 days (range, 29–322 days) and 423 days (range, 34–1243 days) in the TOCE and TOCE + HAIC groups, respectively, with PFS being significantly longer in the combination therapy group as shown in Table 2 and Fig. 2a (*p* = 0.025). DCRs based on assessments prior to each subsequent session were statistically higher for TOCE + HAIC (77.8%) than TOCE (40.0%) (*p* = 0.0025). The representative images of the case treated with TOCE + HAIC that resulted in a complete response are shown in Fig. 3. Although no clear trend for AFP or L3% was observed during the course of either treatment, the increment ratio of DCP during the course of treatment was significantly larger in the monotherapy group, suggesting a better tumor control capability from the combination therapy (*p* < 0.0001, Fig. 2b). However, the overall survival was not significantly different between the monotherapy and combination therapy groups, with median survival times of 706 days and 733 days, respectively (*p* = 0.40, Table 2 and Fig. 2c).

### Safety

The hematological and non-hematological toxicities were evaluated for each treatment session using NCI-CTCAE version 4.0 and are summarized in Table 3. No hematological or non-hematological adverse events of grade 2 or higher were observed. In the TOCE group, the white blood cell and neutrophil counts decreased in two cases (13%), elevated liver enzymes developed in three cases (20%), and nausea was reported in 2 cases (13%). All of these adverse events were categorized as grade 1. In the TOCE + HAIC group, a fever above 37 °C after 5 sessions was present in 2 cases (14%). One of these patients complained of anorexia after 3 sessions (8.3%) and showed hypoalbuminemia after 2 sessions (5.6%). These adverse events were all categorized as grade 1 and were resolved within 2 weeks without any specific treatments. There were no treatment-related deaths in either arm.

**Table 3:** Summary of adverse events

Number of sessions	TOCE				TOCE + HAIC			
	15				36			
	Grade							
Haematological toxicity	1	2	3	4	1	2	3	4
White blood cell decreased	2	0	0	0	0	0	0	0
Neutrophil count decreased	2	0	0	0	0	0	0	0
Platelet count decreased	0	0	0	0	0	0	0	0
Anemia	0	0	0	0	0	0	0	0
Non-haematological toxicity	1	2	3	4	1	2	3	4
AST increased	3	0	0	0	0	0	0	0
ALT increased	3	0	0	0	0	0	0	0
Blood bilirubin increased	0	0	0	0	0	0	0	0
PT-INR increased	0	0	0	0	0	0	0	0
Hypoalbuminemia	0	0	0	0	2	0	0	0
Creatinine increased	0	0	0	0	0	0	0	0
Anorexia	0	0	0	0	3	0	0	0
Nausea	2	0	0	0	0	0	0	0
Vomiting	0	0	0	0	0	0	0	0
Fever	1	0	0	0	5	0	0	0
Diarrhea	0	0	0	0	0	0	0	0
Fatigue	0	0	0	0	0	0	0	0
Alopecia	0	0	0	0	0	0	0	0
Urticaria	0	0	0	0	0	0	0	0
Abdominal pain	1	0	0	0	0	0	0	0

*TOCE* transarterial oily chemoembolization, *TACE* transarterial chemoembolization, *HAIC* hepatic arterial infusion chemotherapy, *PT-INR* prothrombin time-international normalized ratio

**Fig. 4:**
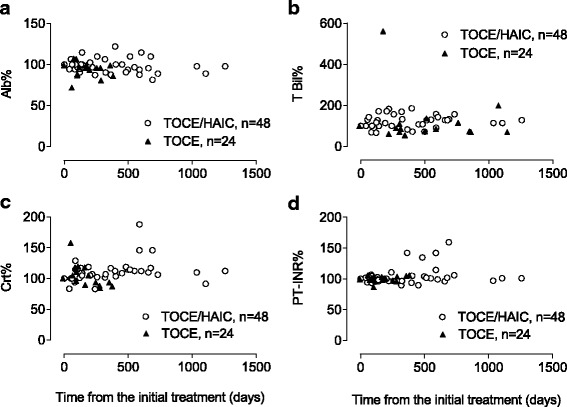
Time dependent changes in serum biomarkers. Serum concentrations of (**a**), albumin (Alb); (**b**), total bilirubin (T Bil); (**c**), creatinine (Crt); and (**d**), prothrombin time-international normalized ratio (PT-INR) are plotted along the course of the treatments. Each value represents a percentage of the serum concentration after treatments versus the value obtained before the initial treatment. The *open circles* and *closed triangles* are data from the combination and monotherapy arms, respectively. The number of plotted data in each arm was shown as “*n*=”

Although none of the serum concentrations of albumin, total bilirubin, creatinine, or prothrombin time-international normalized ratio (PT-INR) showed a significant trend during the treatment course (Fig. 4), a significant reduction in the serum cholinesterase concentration was observed in four out of ten cases receiving the combination therapy (*p* < 0.0001, *r* = 0.86 Fig. 2d and Table 4). On the other hand, the level never dropped below 80% of the initial value in the other cases, including fifteen observation points in nine monotherapy cases, with the exception of one point at which the serum level of cholinesterase reached 68.9% 62 days after initial miriplatin administration. Notably, the two longest survivors, who lived more than three years after the initial combination treatments, never showed a reduction in cholinesterase below 80% of the pretreatment value throughout the entire course of treatment. The TNM and Child-Pugh stages did not significantly influence whether cholinesterase decreased below 80% (*p* = 0.52, *p* = 1.00, respectively).

**Table 4:** Backgrounds of ChE-stable and -worse cases receiving the combination therapy

ChE	Case	Dead/Alive	Period	TNM	Child-Pugh
Stable^a^	1	Dead	334	III	A
	2	Dead	444	III	B
	3	Dead	541	II	B
	4	Alive	695	III	A
	5	Alive	1576	III	A
	6	Alive	1695	II	A
Worse^a^	7	Dead	382	II	A
	8	Dead	700	II	A
	9	Dead	765	III	A
	10	Alive	779	II	B

^a^ The serum concentration of cholinesterase (ChE) got less than 80% of the initial concentration (Worse) or stayed at 80% or higher (Stable)

## Discussion

This study is the first randomized phase II clinical trial to compare the safety and efficacy between miriplatin-TOCE monotherapy and miriplatin-TOCE/DDP-H-HAIC combination therapy in patients with unresectable HCC at BCLC A or B. Because the therapeutic strategy is determined by both hepatic functional reserve and tumor stage, only 30% of HCC cases can benefit from therapies of locoregional control, such as surgical resection, radio frequency ablation, and heavy particle radiotherapy [8, 15, 16]. TACE or sorafenib are options when the tumor stage advances beyond locoregional control. However, these treatments require a relatively higher hepatic functional reserve [17, 18]. Although liver transplantation is another option for patients at that stage, the Milan criteria and donor shortages restrict this indication to only certain cases. However, TOCE and HAIC can be promising alternatives for patients suffering from multiple HCCs, especially when they possess poor hepatic reserve. A randomized phase III trial using zinostatin stimalamer dissolved in lipiodol demonstrated an equivalent therapeutic effect to TACE in patients with multiple HCCs and limited hepatic reserve [5]. Theoretically, in HAIC there is no physical burden due to impaired hepatic blood circulation. This study aimed to clarify the clinical benefit of a novel TOCE/HAIC combination therapy for HCC at BCLC A or B.

Among the various chemotherapeutic agents for TOCE, miriplatin is a unique platinum agent that was developed specifically for HCC. It forms a stable suspension with lipiodol that gradually releases active derivatives in situ, which enables a higher localized concentration while circumventing spillover into systemic circulation [19]. Miriplatin has demonstrated antitumor effects and a promising safety profile [7, 20, 21]. In terms of chemotherapeutic agents for HAIC, platinum agents appear to be most effective. In a multicenter phase II study that enrolled unresectable HCC cases [10], the response rate for cisplatin was 33.8%, whereas the response rates were 15%–20% for epirubicin [9] and mitomycin C [8]. Because miriplatin shows no cross-resistance with different generations of platinum agents [6, 22, 23], we examined double platinum treatments and anticipated additive effects of miriplatin and DDP-H, which currently provides the highest concentration of CDDP. The phase I study of this combination therapy showed no dose-limiting toxicity at a maximum dose of 120 mg/body for TOCE [24] and 65 mg/m^2^ for HAIC. Importantly, no systemic platinum release from miriplatin-TOCE was found [24].

Consistent with the phase I study, the miriplatin-TOCE/DDP-H-HAIC combination therapy could be repeated safely with no severe adverse events. In fact, the number of repetitive sessions was significantly higher in the combination group, leading to significantly higher PFS and DCR. Consistently, DCP revealed a significantly slower increment in the combination therapy group. All these results strongly suggest that the combination therapy has better anti-cancer effects than the monotherapy. However, overall survival was not significantly different between the two types of treatments. As scoring systems that integrate both anatomical tumor extent and functional hepatic reserve have a better capability to stratify survival than TNM stage, the reason for the inconsistency between PFS/DCR and OS may be due to the reciprocal impairment of functional hepatic reserve in the combination therapy. The combination therapy may have the potential to alleviate cancer progression, but at the same time may reduce functional hepatic reserve. Actually, the combination therapy induced a linear reduction of cholinesterase during the course of treatment; however, it happened in specific cases. It did not occur in cases that demonstrated the longest survival time. If we can distinguish the cases that can tolerate these combination treatments, the combination therapy would show preferable results for OS, too. Altered cholinesterase during the course of treatment may provide valuable information to differentiate the cases that would benefit from combination therapy.

The limitations of this study were that the number of enrolled patients was small and that miriplatin and DDP-H are not available in many countries. An additional study should be conducted with a larger cohort to confirm the efficacy of TOCE + HAIC and to develop a practical method to predict the cases that will tolerate the combination therapy.

## Conclusions

In conclusion, double platinum chemotherapy using miriplatin-TOCE and cisplatin-HAIC can be safely administered, and it resulted in better disease control for intermediate-stage HCC patients at BCLC A to B. This therapy can be repeated without severe toxicity, leading to higher disease control rates than miriplatin-TOCE monotherapy. An additional study is needed confirming the results in a larger cohort, and the efforts are necessary to establish a marker predicting the cases tolerable for the combination therapy. However, our study showed that miriplatin-TOCE/DDP-H-HAIC combination therapy can contribute to an improved prognosis in HCC patients.

## Abbreviations

AFP: Alpha-fetoprotein
CDDP: Cisplatin
CI: Confidence interval
DCP: Des-gamma-carboxy prothrombin
DCR: Disease control rate
DDP-H: Cisplatin powder
HAIC: Hepatic arterial infusion chemotherapy
HCC: Hepatocellular carcinoma
L3%: Percentage of fucosylated AFP versus total AFP
NCI-CTCAE: National Cancer Institute Common Terminology Criteria for Adverse Events
OS: Overall survival
PFS: Progression free survival
TACE: Transarterial chemoembolization
TOCE: Transarterial oily chemoembolization
